# Elucidating the Impact of High-Temperature *Daqu* on Base Baijiu of Sauce-Flavor Baijiu: From Key Aroma Compounds to Microbial Origins

**DOI:** 10.3390/foods15071124

**Published:** 2026-03-24

**Authors:** Peng Chen, Shiming Shen, Liangcai Lin, Qijing Liu, Cuiying Zhang, Cheng Zhong

**Affiliations:** State Key Laboratory of Food Nutrition, Safety Key Laboratory of Industrial Fermentation Microbiology (Ministry of Education), Tianjin University of Science and Technology, Tianjin 300457, China; cp18322484685@163.com (P.C.); s13021895619@163.com (S.S.); lclin@tust.edu.cn (L.L.); qijingliu@tust.edu.cn (Q.L.); cyzhangcy@tust.edu.cn (C.Z.)

**Keywords:** high-temperature *Daqu*, base baijiu, key flavor compounds, microbial

## Abstract

*Jiaomian* base baijiu is an important seasoning liquor used in the blending of sauce-flavor baijiu, yet the mechanism underlying its flavor formation remains insufficiently understood. Moreover, the specific contribution of high-temperature *Daqu* (*HTDQ*) to the flavor profile of *Jiaomian* base baijiu has not been clearly defined. Therefore, this study compared the aroma profiles and flavor compounds of *Jiaomian* and *Chuntian* base baijiu. *Jiaomian* base baijiu displayed stronger Qu-aroma and floral–fruity notes, with six differential flavor markers (VIP > 1) identified, including ethyl acetate, acetaldehyde, and n-propanol. Further analysis showed that yellow *HTDQ* exhibited greater inner–outer heterogeneity in aroma and flavor profiles than white and black *HTDQ*. It also contained the highest concentration of flavor compounds and exerted the strongest influence on the flavor of *Jiaomian* base baijiu. By comparing the flavor compounds of *HTDQ* and base baijiu, 14 key compounds were identified that mediate the influence of *HTDQ* on the flavor of *Jiaomian* base baijiu. These compounds were primarily formed during the early to middle stages of *HTDQ* fermentation. Correlation analysis further indicated that microorganisms during *HTDQ* fermentation were predominantly positively correlated with the key flavor compounds. Among them, *Thermoactinomyces*, *Byssochlamys*, *Kazachstania*, *Leiothecium*, and *Trichothecium* showed the closest associations—positively correlated with compounds such as 1-nonanol and furfuryl alcohol, and negatively correlated with isovaleric acid. Finally, KEGG enrichment analysis of the flavor compounds suggested that Beta Oxidation of Very Long-Chain Fatty Acids, Mitochondrial Beta-Oxidation of Short-Chain Saturated Fatty Acids, and Fatty Acid Biosynthesis are key pathways involved in the formation of these flavor substances. In summary, this study clarifies the key flavor compounds through which different *HTDQ* types influence base baijiu flavor, reveals the microbial origins and metabolic pathways of these key flavor compounds, and provides a theoretical basis for regulating *HTDQ* production and improving the quality of base baijiu.

## 1. Introduction

Baijiu is recognized as one of the world’s six major distilled spirits, with sauce-flavor baijiu representing a quintessential archetype within this category [1,2]. It is characterized by a distinct soy sauce aroma and a persistent fragrance that lingers in an emptied glass, contributing to its widespread appreciation among consumers both in China and abroad. The production of sauce-flavor baijiu involves seven successive fermentation rounds, each yielding a base baijiu with a unique sensory profile [3,4]. Additionally, during the same production cycle, variations in processing techniques and fermentation positions yield three distinct types of base baijiu: *Jiaomian*, *Chuntian*, and *Jiaodi* base baijiu [5,6]. *Jiaomian* base baijiu is distilled from fermented grains in the upper layer of the pit, exhibiting strong soy sauce and *Qu* aromas. *Chuntian* base baijiu, obtained from the middle layer, presents a harmonious blend of soy sauce aroma and mild sweetness. *Jiaodi* base baijiu, derived from the lower layer, carries a distinct cellar-floor-derived aroma [5,6,7]. Gong et al. (2023) conducted a systematic study on the flavor profiles of three base baijiu of sauce-flavor baijiu, identifying 37 key differential compounds, including critical aroma-active compounds such as butyric acid, butyl 2-methylbutyrate, and ethyl caproate, which significantly contribute to sweet, fruity, pit mud, sauce aroma, and alcoholic aromas [8]. The flavor characteristics of different types of base baijiu are influenced by factors such as the high-temperature *Daqu* (*HTDQ)* used, production processes, and pit mud quality [5,6,8]. Wang (2025) [5] compared the effects of manual and mechanical ventilation on the flavor profiles of *Jiaomian* and *Chuntian* base baijiu. They found that while *Chuntian* baijiu exhibited minimal flavor variation between ventilation methods, *Jiaomian* baijiu showed more pronounced differences. Specifically, mechanical ventilation significantly elevated the concentrations of 2,3-dimethyl-5-ethylpyrazine, 2,3-dimethylpyrazine, tetramethylpyrazine, and ethyl benzoate in *Jiaomian* baijiu [5]. Furthermore, *HTDQ* and pit mud represent additional key factors shaping the distinct flavor characteristics of various base baijiu types of sauce-flavor baijiu [6]. During production, the amount of *HTDQ* used for *Jiaomian* base baijiu is 1.5 to 2 times greater than that for *Chuntian* base baijiu, which imparts a more intense Qu aroma to the former. Blending base baijiu with different sensory profiles is a critical technique in baijiu production and is essential for enhancing overall product quality [9]. Consequently, the production of *Jiaomian* base baijiu holds particular significance for subsequent blending processes aimed at intensifying the *Qu* aroma in the final baijiu.

The flavor characteristics of different types of base baijiu of sauce-flavor baijiu are closely linked to the quality of *HTDQ* [7]. *HTDQ* serves not only as a saccharifying and fermenting agent but also as a crucial source of raw materials and flavor compounds, significantly shaping the flavor profile of the base baijiu [10]. It acts as a core driver and key origin of the characteristic flavor compounds in sauce-flavor baijiu [11]. During fermentation, the microorganisms and enzymes in *HTDQ* generate a large number of flavor compounds. Simultaneously, the complex aroma components inherent in the *HTDQ* itself are transferred into the base baijiu through distillation and other processes, imparting to sauce-flavor baijiu a composite aroma highly reminiscent of *HTDQ*. This aroma, termed “*Qu*-aroma” by the academic community, is a key sensory indicator for assessing product quality grade [12,13,14,15]. The *Qu*-room fermentation stage is critical for determining the quality of *HTDQ*. During this phase, the *HTDQ* enriches various functional microbial communities (including molds, bacteria, and yeasts), contains hydrolytic enzymes such as amylases and proteases, nutritional substrates like carbohydrates and proteins, as well as various aroma precursors and characteristic volatile components. These elements collectively provide the essential material foundation, microorganisms, and enzymes for the formation of the baijiu’s distinctive flavor [10,16,17]. Due to spatial heterogeneity during *Qu*-room fermentation, three types of *HTDQ* are produced: white *HTDQ*, yellow *HTDQ*, and black *HTDQ* [17]. These types differ in physicochemical properties, flavor compounds, and microbial composition. Research by Shi et al. indicate that white *HTDQ* exhibits the highest saccharification and liquefaction power, black *HTDQ* shows the highest cellulase and neutral protease activity, while yellow and black *HTDQ* contain the highest levels of flavor compounds [17].

The formation of flavor compounds in *HTDQ* is closely associated with its microbial composition [18]. Jin et al. (2019) [19] investigated the aroma compounds and microbial communities at different locations within *HTDQ*, along with their correlations. They found that microbial interactions were stronger in the core of the *HTDQ* than in its surface layer. *Bacillus* and *Lactobacillus* showed positive correlations with pyrazines and esters, respectively, while *Aspergillus* was linked to the metabolism of pyrazines, esters, and aromatic compounds [19]. Peng Wang et al. (2017) [20] used *Bacillus licheniformis*, a dominant microbial strain in *HTDQ*, as a starter to study its impact on the microbial community and metabolic products of *HTDQ*. Their research confirmed the significant role of *Bacillus licheniformis* in shaping the *HTDQ* microbiome and aroma profile [20]. He et al. (2019) [21] investigated the effect of fortified strains (*Bacillus velezensis* and *Bacillus subtilis*) on the aroma compounds of *HTDQ*. They observed that the addition of these strains promoted the growth of Bacillus, *Lactobacillus*, and *Candida*, enhanced the saccharification, liquefaction, and esterification capabilities of *HTDQ*, and significantly increased the contents of esters, pyrazines, and alcohols in the fortified *HTDQ* [21].

Currently, research on *HTDQ* has primarily focused on its physicochemical properties, microbial composition, and flavor compounds, yet has not sufficiently elucidated its impact on the quality of base baijiu. Therefore, this study selected *Jiaomian* base baijiu and *Chuntian* base baijiu as samples. The aroma characteristics and differences in flavor compound profiles between these two types of base baijiu were analyzed through sensory evaluation and flavor detection techniques. Subsequently, the aroma characteristics and flavor compounds of different types of *HTDQ* were analyzed. At the level of flavor compounds, the material basis through which *HTDQ* influences the flavor of base baijiu was investigated, and key flavor compounds were identified. Finally, the correlations between key flavor compounds and microorganisms during the *HTDQ* fermentation process, as well as the related formation pathways, were further analyzed. Through the above research, this paper clarifies the material basis by which *HTDQ* influences the flavor characteristics of base baijiu and elucidates the formation pathways of key flavor compounds. This study holds significant importance for the production and application of *HTDQ* and is beneficial for the high-quality production of base baijiu.

## 2. Materials and Methods

### 2.1. Samples

A total of 24 base baijiu samples were collected, including 9 *Jiaomian* base baijiu (JMJ) samples and 15 *Chuntian* base baijiu (CTJ) samples. Compared to CTJ, the production process of JMJ involves 1.5 to 2 times the amount of *HTDQ* used in fermentation [7,8].

The *HTDQ* used in this study was provided by Guizhou Guotai Digital Intelligence Distillery Group Co., Ltd. (Maotai Town, Guizhou Province, China; produced in 2023). During the *HTDQ* fermentation process, samples were collected at four time points: 7 d, 14 d, 21 d, 28 d, 35 d, and 40 d (end of fermentation). At each time point, samples were taken separately from the upper, middle, and lower layers of the *Qu* room and then mixed to form one representative sample.

For the finished *HTDQ* products, three types were collected: yellow *HTDQ*, white *HTDQ*, and black *HTDQ*, with three replicates for each type. For each replicate, the surface layer and inner layer were separated. Subsequently, three samples of the same type and from the same layer were pooled into one representative sample for subsequent sensory evaluation and flavor compound analysis.

### 2.2. Sensory Evaluation

The sensory evaluation panel consisted of six trained assessors (five males and five females). The panel first conducted a comprehensive descriptive analysis of the aroma profiles of both base baijiu and *HTDQ*. Based on preliminary sensory profiling and references in the literature [11], six key aroma descriptors were selected for base baijiu evaluation: *Qu* aroma, sauce aroma, floral and fruity aroma, grain aroma, cellar aroma, and caramelized aroma. For *HTDQ* evaluation, seven descriptors were employed: *Qu* aroma, sauce aroma, wheat aroma, *Douchi* aroma, caramel aroma, musty odor, and sweet aroma.

Each aroma attribute was evaluated using a five-point intensity scale (0 = none, 1 = weak, 3 = moderate, 5 = strong). Each sample was assessed in triplicate by every panelist, and the final scores were averaged across all panelists and replicates to establish the aroma fingerprints of the baijiu and *HTDQ* samples.

### 2.3. Analysis of Flavor Compounds in Samples

The flavor compounds in the base baijiu samples were analyzed using GC-FID and HS-SPME-GC-MS, while those in the *HTDQ* samples were analyzed using HS-SPME-GC-MS. The specific methods are detailed below.

GC-FID analysis: 8 mL of ultrapure water and 3.0 g of Daqu powder were placed in a 20 mL headspace bottle and incubated at 80 °C with agitation at 500 r/min for 15 min. Subsequently, 200 μL of headspace gas was automatically injected. GC conditions: a column temperature of 60 °C, with ultrapure nitrogen (≥99.999%) as the carrier gas flowing at rates of 2 mL/min (0–2 min), 2–10 mL/min (2–10 min), 10–100 mL/min (10–25 min), and 100 mL/min (25–30 min), over a total detection time of 30 min. Detector: Hydrogen Flame Ionization Detector (FID); carrier gas: nitrogen; Hydrogen flow rate: 35 mL/min; Airflow rate: 350 mL/min; Inlet temperature: 200 °C; Detector temperature: 250 °C; Injection volume: 1 µL; Split ratio: 20:1; Column: HP-FFAP (30 m × 0.5 mm × 1.00 µm); Temperature program: initial temperature 37 °C held for 7 min; increased at 5 °C/min to 80 °C; then at 10 °C/min to 100 °C, held for 4 min; finally increased at 15 °C/min to 200 °C, held for 7 min.

HS-SPME-GC-MS analysis for flavor compounds in *HTDQ* and base baijiu: The method was adapted from previous studies with appropriate modifications [22]. Precisely 2 mL of base baijiu was transferred into a 20 mL headspace vial. Then, 10 mL of ultrapure water, 2.0 g of NaCl, and 50 µL of internal standard solution (prepared by diluting 50 µL of 2-ethylbutyric acid to 50 mL with 60% anhydrous ethanol) were added. The sample was equilibrated at 50 °C for 10 min. A 50/30 µm DVB/CAR/PDMS fiber was inserted into the vial for headspace adsorption for 50 min. After extraction, the fiber was immediately transferred to the GC-MS injection port and desorbed at 250 °C for 5 min prior to GC-MS analysis.

Analysis and identification of aroma compounds: Analysis was performed using a GC Ultra gas chromatograph coupled with a DSQ II mass spectrometer (Thermo Electron Corp., Waltham, MA, USA). The mass spectrometer was operated in electron impact (EI) mode at 70 eV, with a full scan range of 30–550 amu. Separation was achieved using a DB-FFAP column (30 m × 0.25 mm × 0.25 µm, Agilent Technology, Santa Clara, CA, USA). The inlet temperature was set at 250 °C with a split ratio of 10:1. High-purity nitrogen (99.999%) was used as the carrier gas at a flow rate of 1.5 mL/min. The oven temperature program was as follows: initial temperature 50 °C, held for 2 min; increased at 5 °C/min to 190 °C; then at 10 °C/min to 230 °C and held for 10 min. The auxiliary heater temperature was set at 250 °C. Compound identification was performed by matching mass spectrometry with the NIST 15 database, and the accuracy of identification was verified by comparing the retention index of the sample with the retention index reported in the literature. Only compounds with a matching factor greater than 800 were considered. To verify the identification, a series of n-alkanes (C8–C40), analyzed under the same chromatographic conditions were used to calculate the retention index (RI) of each compound. The calculated RI was then compared with the reported RI in the NIST database and published literature [23,24,25,26,27,28,29,30,31,32,33]. The maximum deviation for identification confirmation is 30.

### 2.4. High-Throughput Sequencing of HTDQ Samples

After extraction of genomic DNA, the quality and integrity of the extracted DNA were evaluated by 1% agarose gel electrophoresis. For microbial community analysis, the V3–V4 region of bacterial DNA was amplified using the universal bacterial primers 520F/820R, while the target region of fungal DNA was amplified with the universal fungal primers ITS/ITS5. The amplified sequences were sequenced on the MiSeq platform (Illumina), and high-quality sequences were obtained through subsequent data processing (including filtering of low-quality reads and removal of chimeric sequences). All high-quality sequences were clustered into Operational Taxonomic Units (OTUs) at a 97% sequence similarity cutoff. Bioinformatics statistical analyses were performed based on the OTU classification results. Finally, BLAST (version 2.2.28+) alignment was conducted against the NCBI (National Center for Biotechnology Information) database to annotate the taxonomic information of the OTUs.

### 2.5. Statistical Analysis and Visualization

All statistical analyses were performed with three independent replicates. Data visualization and analysis were conducted using a combination of specialized tools and platforms. Heatmaps were generated using TBtools (version 2.39.0). Venn diagrams were created on the Lianchuan Biocloud Platform. Data processing, multivariate statistical analysis, and correlation calculations were executed using Python (version 3.12.7) within the Jupyter Notebook (version 7.2.2) environment, utilizing specialized libraries such as Pandas, Scikit-learn, and NumPy. The resulting correlation networks were visualized using Gephi software (version 0.9.7).

## 3. Results and Discussion

### 3.1. Analysis of Aroma Characteristics and Flavor Composition of Different Base Baijiu of Sauce-Flavor Baijiu

**Figure 1:**
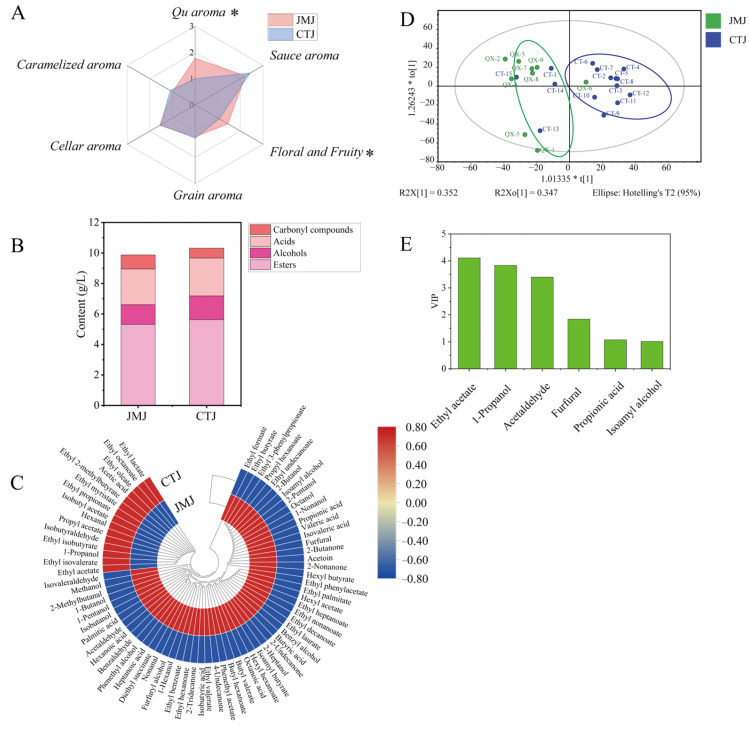
Analysis of differences in aroma characteristics and flavor substances among different types of sauce-flavor base liquors. (**A**) Aroma profiles (* indicate significant differences, *p* < 0.05), (**B**) contents of different flavor categories, and (**C**) heatmap of flavor composition in *Jiaomian* and *Chuntian* base liquors; OPLS-DA analysis (**D**) and identification of marker compounds (**E**) responsible for flavor differences between the two base liquors.

The aroma characteristics and flavor compound profiles vary across different types of base baijiu of sauce-flavor baijiu, which significantly impacts the subsequent blending process and ultimately affects the quality of the final Baijiu product [7,34]. Therefore, sensory evaluation was first employed to analyze the aroma characteristics of *Jiaomian* base baijiu (JMJ) and *Chuntian* base baijiu (CTJ). As shown in Figure 1A, the *Qu* aroma and floral/fruity notes of JMJ were significantly stronger than those of CTJ. The aroma profile of base baijiu is closely linked to its flavor composition. Therefore, a combination of analytical techniques, including GC-FID and HS-SPME-GC-MS was used to detect volatile compounds in the two types of base baijiu. A total of 72 flavor compounds were identified, comprising 33 esters, 15 alcohols, 10 acids, and 14 carbonyl compounds (aldehydes and ketones) (Figure 1B,C). Esters and acids were the predominant flavor components common to both base baijiu, followed by alcohols and carbonyl compounds. Further comparison of the flavor composition revealed that 15 compounds, such as ethyl acetate, 1-propanol, and ethyl isovalerate, were more abundant in CTJ. In contrast, 57 compounds, including ethyl formate, ethyl butyrate, and phenethyl alcohol, were found at higher levels in JMJ (Figure 1C). These flavor substances have important contributions to the aroma characteristics of base liquor. For example, phenylethanol, which has a typical rose fragrance, is an important flower flavor compound in Baijiu [35]. Ethyl butyrate is mainly produced by esterification reactions during microbial fermentation and is one of the symbolic components of Luzhou flavor Baijiu. In the upper fermented grains of Nongxiangxing baijiu, the content of ethyl butyrate is often low, which directly affects the quality of the upper base liquor [36].

The OPLS-DA model revealed a clear separation between the two baijiu types, corroborating their distinct flavor profiles (Figure 1D). From this model, six compounds were screened as critical markers for differentiation: ethyl acetate, 1-propanol, acetaldehyde, furfural, propionic acid, and isoamyl alcohol. These compounds met the criteria of VIP scores > 1.0 and *p*-values < 0.05 (Figure 1E) [37]. Part of these findings is consistent with the research by Yang et al., who also identified 1-propanol and acetaldehyde as key markers differentiating JMJ and CTJ, with 1-propanol higher in CTJ and acetaldehyde higher in JMJ [7]. These flavor compounds significantly contribute to the differential aroma characteristics of the base baijiu, and their concentration differences between the two baijiu types are a major reason for their distinct aroma profiles [38]. This is because, on one hand, these compounds directly contribute to the flavor profile. For example, ethyl acetate imparts floral and fruity notes to both base baijiu [38]. On the other hand, flavor compounds can also influence the perception of other compounds’ aromas. For instance, studies by Han et al. have shown that 1-propanol can suppress the volatility of esters like ethyl acetate and ethyl butyrate, thereby weakening floral and fruity notes [38]. This might explain why CTJ, despite having higher ethyl acetate content, exhibits weaker floral and fruity aromas. Furthermore, these differential flavor compounds also affect the taste of Baijiu. For example, 1-propanol is one of the key bitter compounds in Baijiu, and excessive amounts can negatively impact base liquor quality [4].

### 3.2. Analysis of Aroma Characteristics of Different Types of HTDQ

**Figure 2:**
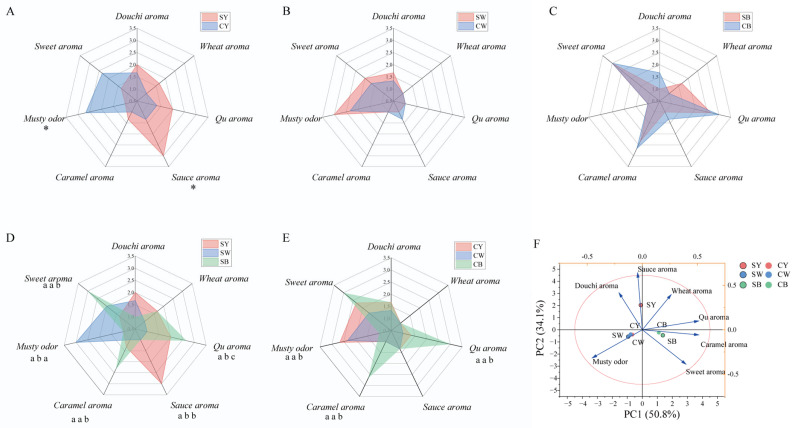
Aroma characteristic analysis of different types of *HTDQ*. Analysis of aroma profiles in the inner and surface layers of (**A**) white *HTDQ*, (**B**) yellow *HTDQ*, and (**C**) black *HTDQ*; comparison of aroma characteristics in the (**D**) surface layer and (**E**) inner layer among the three types of *HTDQ*, along with (**F**) PCA. (S: surface layer; C: core layer). * and letters in (**A**–**E**) indicate significant differences (*p* < 0.05).

In the production of Baijiu, the dosage of *HTDQ* used for *Jiaomian* base Baijiu is typically higher than that for *Chuntian* base Baijiu, resulting in a greater transfer of flavor compounds from *HTDQ* into the former. *HTDQ* itself is primarily categorized into types such as yellow, white, and black *HTDQ*, each exhibiting distinct aroma characteristics and flavor profiles [13,39]. All of these types serve as crucial sources for shaping the flavor and influencing the quality of base Baijiu [13,39]. To systematically elucidate the mechanism by which different types of *HTDQ* affect the flavor of base Baijiu, this study first analyzed the aroma characteristics and flavor composition of various *HTDQ* types and their different sections. As shown in Figure 2A, the surface layer of yellow *HTDQ* (SY) exhibited predominant sauce, *Qu*, and *Douchi* aroma notes, while its inner core (CY) presented predominant moldy and sweet aromas. Specifically, the sauce aroma was significantly higher in the surface layer, whereas the musty aroma was significantly higher in the inner core of yellow *HTDQ* (CY). Both the surface and inner core sections of white *HTDQ* (SW and CW) displayed a predominant musty aroma (Figure 2B). Both sections of black *HTDQ* (SB and CB) were characterized by pronounced *Qu*, caramel, and sweet aromas (Figure 2C). No significant differences were observed between the surface and inner core sections of white and black *HTDQ*. These results indicate that, compared to yellow *HTDQ*, the aroma profiles of black and white *HTDQ* are more homogeneous, while yellow *HTDQ* exhibits stronger heterogeneity, contributing to its overall richer and more complex aroma profile.

To further compare the aroma differences among the different types of *HTDQ*, Figure 2D indicates that the outer layer of black *HTDQ* exhibited significantly stronger *Qu* aroma, caramel aroma, and sweet aroma compared to the outer layers of white *HTDQ* and yellow *HTDQ*. Therefore, increasing the proportion of outer-layer black *HTDQ* may enhance the *Qu*, caramel, and sweet characteristics in the base baijiu. Additionally, the outer layer of yellow *HTDQ* showed a significantly stronger sauce aroma than the outer layers of white *HTDQ* and black *HTDQ*, suggesting that increasing the proportion of yellow *HTDQ* could strengthen the sauce aroma in the base baijiu. Comparing the aroma characteristics of the inner cores of the three types of *HTDQ* (Figure 2E), the inner black *HTDQ* demonstrated significantly higher *Qu* and caramel aromas than white and yellow *HTDQ*, while its musty aroma was significantly lower than that of white and yellow *HTDQ*. In summary, the aroma characteristics vary among different types and sections of *HTDQ*, and their proportional usage can influence the flavor profile of the base baijiu.

Finally, Principal Component Analysis (PCA) was employed to analyze the overall aroma characteristics of the different types and sections of *HTDQ*. The results revealed a clear separation among the three types of *HTDQ*, indicating distinct aroma profiles (Figure 2F). Specifically, the outer and inner black *HTDQ* clustered together, suggesting similar overall aroma characteristics. The inner white *HTDQ*, outer white *HTDQ*, and inner yellow *HTDQ* formed another cluster, sharing similar aroma traits. The inner and outer yellow *HTDQ* were distantly separated, reflecting the strong spatial heterogeneity in the aroma profile of yellow *HTDQ*. In conclusion, among the three types of *HTDQ*, yellow *HTDQ* exhibited the greatest aroma heterogeneity between its sections, while white and black *HTDQ* were relatively uniform. Compared to the inner sections, the aroma differences in the outer layers of the three *HTDQ* types were more pronounced, primarily manifested in Qu aroma, sauce aroma, caramel aroma, sweet aroma, and musty aroma. These findings underscore that the use of different types and sections of *HTDQ* in the brewing process is a key factor influencing the quality of the base baijiu.

### 3.3. Analysis of Flavor Composition in Different HTDQ Types and Tracing Key Compounds to Their Contribution to Base Baijiu Flavor

**Figure 3:**
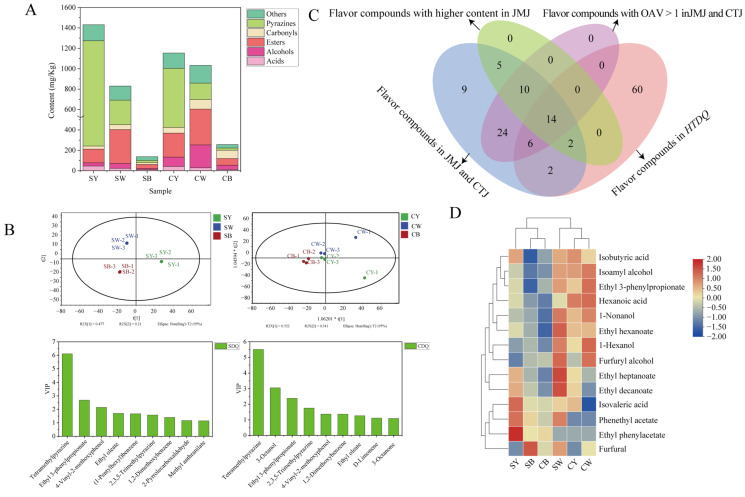
Analysis of flavor composition and differences among different types of *HTDQ*. (**A**) Flavor composition of different types of *HTDQ*, (**B**) differential analysis, (**C**) Venn diagram illustrating the overlap of flavor compounds between *HTDQ* and base liquor, and (**D**) key flavor compounds derived from *HTDQ* that shape the flavor profile of base baijiu.

The differences in aroma characteristics among different types and sections of *HTDQ* are primarily attributed to their volatile flavor compounds, which constitute the substance basis for influencing the flavor profile of the base baijiu [40,41,42]. To analyze this material basis, the flavor composition of different *HTDQ* types was further investigated using HS-SPME-GC-MS. A total of 84 flavor compounds were identified, including 17 esters, 12 alcohols, 11 pyrazines, 8 acids, and 8 aldehydes. The content of these flavor compounds in the three *HTDQ* types was first compared. As shown in Figure 3A, yellow *HTDQ* exhibited the highest total content, followed by white *HTDQ*, with black *HTDQ* having the lowest, regardless of the section (outer or inner). In both yellow and white *HTDQ*, pyrazines were the most abundant class, followed by esters, in both sections. In black *HTDQ*, esters and carbonyl compounds were the most prevalent. OPLS-DA was subsequently applied to compare the overall flavor profiles of the three *HTDQ* types from different sections. As shown in Figure 3B, samples from different sections of the three *HTDQ* types were located in distinct quadrants and were widely separated, indicating substantial flavor differences. Based on the criteria of VIP > 1 and *p* < 0.05, nine marker compounds were identified as key for discriminating the outer sections of the three *HTDQ* types. Similarly, nine marker compounds were identified for discriminating their inner sections. Six common discriminatory markers were found: tetramethylpyrazine, ethyl 3-phenylpropionate, 2,3,5-trimethylpyrazine, 4-vinyl-2-methoxyphenol, 1,2-dimethoxybenzene, and ethyl oleate.

To investigate the connection between *HTDQ* and base baijiu in terms of flavor composition, a comparative analysis of their volatile profiles was conducted. As shown in Figure 3C, a total of 24 common flavor compounds were identified across different types of base baijiu and *HTDQ*. Among these, 14 key compounds—including furfural, isoamyl alcohol, and phenethyl acetate—were present at significantly higher concentrations in *Jiaomian* base baijiu than in *Chuntian* base baijiu, all with Odor Activity Values (OAVs) > 1. These 14 compounds are preliminarily regarded as the important substance basis through which *HTDQ* influences the distinct flavor characteristics of different base baijiu. After being introduced into the base baijiu from *HTDQ*, these compounds significantly contribute to its aroma profile. The amount of *HTDQ* used in *Jiaomian* base baijiu is approximately 1.5–2 times that in *Chuntian* base baijiu, which also forms part of the material basis for *Jiaomian* base baijiu’s stronger Qu aroma and floral/fruity notes [43,44,45,46]. For instance, compounds such as ethyl phenylacetate, phenethyl acetate, ethyl 3-phenylpropionate, ethyl hexanoate, and 1-nonanol can impart rose, honey, sweet, and fruity characteristics to sauce-flavor base baijiu [43,44,45,46]. Additionally, aroma-active compounds such as furfural (bready), isovaleric acid (sour), isobutyric acid (sour, pungent), hexanoic acid (sweaty), isoamyl alcohol (bitter almond), and 1-hexanol (green) contribute to the more complex and layered *Qu* aroma profile of *Jiaomian* base baijiu. These findings align with previous studies, in which isovaleric acid has also been identified as an important Qu-aroma compound [45]. Further analysis revealed distinct distribution patterns for the 14 key flavor compounds across *HTDQ* types. Specifically, compounds such as furfural, ethyl phenylacetate, phenethyl acetate, and isovaleric acid were more abundant in black *HTDQ* and the surface layer of yellow *HTDQ*, whereas isobutyric acid, isoamyl alcohol, ethyl 3-phenylpropionate, hexanoic acid, 1-nonanol, ethyl hexanoate, and 1-hexanol were enriched in white *HTDQ* and the inner core of yellow *HTDQ*. In summary, yellow *HTDQ*, with the highest diversity and content of flavor compounds, exerted the predominant influence on the flavor profile of *Jiaomian* base baijiu, while black *HTDQ* had a relatively minor impact. Therefore, the rational selection and utilization of specific *HTDQ* types and sections are crucial for the precise regulation and design of base baijiu flavor characteristics [16].

### 3.4. Changes in Key Flavor Compounds and Microbial Communities During HTDQ Fermentation

**Figure 4:**
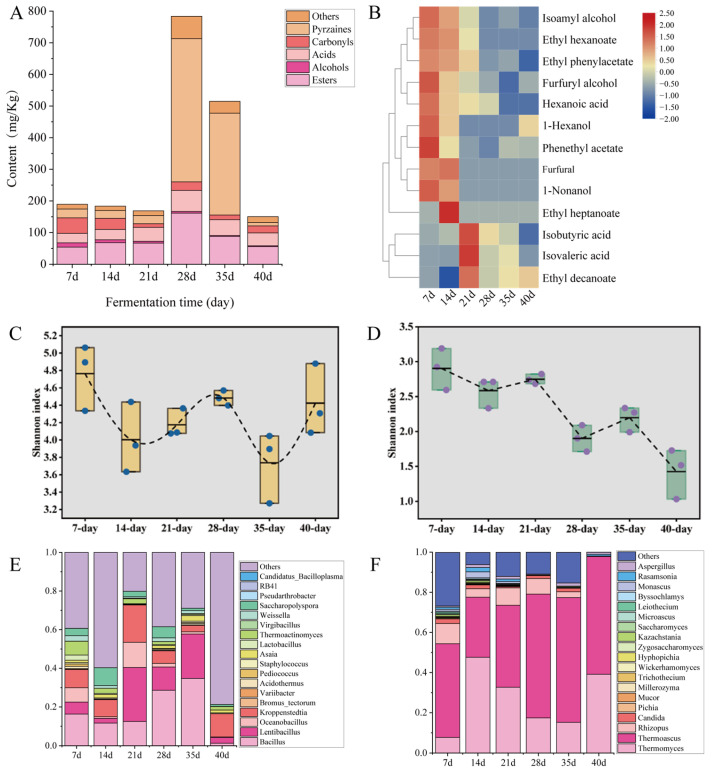
Dynamics of microbial communities and flavor compounds during the fermentation of *HTDQ*. (**A**) Composition of flavor compounds during *HTDQ* fermentation. (**B**) Variation in key flavor compounds in *HTDQ*. (**C**) Changes in bacterial and (**D**) fungal Shannon indices during fermentation. (**E**) Top 20 bacterial and (**F**) fungal taxa identified in *HTDQ*.

The *Qu*-room fermentation stage is critical for the generation of flavor compounds in *HTDQ*, a process closely linked to microbial metabolic activities [33,46,47]. Therefore, we further analyzed the flavor compound profiles and microbial communities of *HTDQ* at different fermentation time points. As shown in Figure 4A, the main flavor compounds produced during *HTDQ* fermentation include esters, acids, carbonyl-containing compounds, and pyrazines. Among them, esters represent one of the major flavor components in *HTDQ*. Their content gradually increased from day 7 to day 28 and then decreased from day 28 to day 40. This increase phase coincided with the rising temperature during fermentation (Figure S1). As precursors for ester synthesis, acids exhibited a trend largely consistent with that of esters, indicating a close relationship between their contents [48]. Carbonyl-containing compounds declined in content from day 7 to day 21, likely because these compounds served as intermediates or precursors in the Maillard reaction, contributing to the formation of pyrazines. Pyrazine content peaked between day 28 and day 35, a period characterized by higher fermentation temperatures that may have promoted the Maillard reaction [49]. Further analysis of the variation trends of key flavor compounds during fermentation identified a total of 13 key flavor substances. Among these, 10 compounds—including isoamyl alcohol, ethyl hexanoate, and ethyl phenylacetate—showed higher levels during days 7–14 of fermentation, whereas 3 compounds—isobutyric acid, isovaleric acid, and ethyl decanoate—reached their peak concentrations on day 21. In summary, it can be concluded that the 13 key flavor compounds are mainly formed during the early to middle stages of *HTDQ* fermentation. Notably, ethyl 3-phenylpropionate was not detected in any *HTDQ* samples across the fermentation time points. This could be due to its content falling below the detection limit during fermentation or because this compound primarily forms during the post-fermentation storage stage. In future studies, we will continue to investigate the composition and evolution of flavor compounds during the storage of *HTDQ*.

In *HTDQ* fermentation, the formation of flavor compounds is closely linked to microbial activity [50]. Therefore, we further analyzed the diversity and composition of the microbial community during fermentation. Analysis of the Shannon index of the microbial community during fermentation revealed distinct dynamics. As shown in Figure 4C, the Shannon index of the bacterial community exhibited a fluctuating “W”-shaped declining trend overall, indicating instability in both the richness and evenness of bacterial populations throughout the process. Conversely, the Shannon index for the fungal community demonstrated a gradual decreasing trend, suggesting a steady reduction in fungal richness and evenness (Figure 4D). These results collectively indicate divergent dynamics in the richness and diversity of bacterial and fungal communities during *HTDQ* fermentation. Further analysis of the microbial composition revealed dominant bacterial genera, including *Bacillus, Lentibacillus*, *Oceanobacillus*, *Kroppenstedtia*, *Saccharopolyspora*, *Thermoactinomyces*, *Weissella*, and *Asaia* (Figure 4E). Among these, the relative abundance of Bacillus initially decreased from day 7 to day 21, then increased from day 21 to day 35, peaking on day 35 (34.77%) and reaching its lowest level (1.35%) by the end of fermentation. Under high-temperature conditions, *Bacillus* contributes to characteristic aromas such as roasted, creamy, and soy-sauce-like notes through the production of pyrazines, acetoin, and guaiacol [51]. Similarly, *Kroppenstedtia* and *Lentibacillus* exhibited an initial decline followed by an increase. *Kroppenstedtia* reached its highest relative abundance (19.32%) on day 21, while *Lentibacillus* peaked (ranging from 12.02% to 27.96%) between days 28 and 35. *Thermoactinomyces* was relatively abundant during the early stage (days 7–14) but declined as fermentation progressed. Analysis of the dominant fungal taxa identified high-abundance genera, including *Thermoascus*, *Thermomyces*, and *Rhizopus* (Figure 4F). *Thermomyces*, one of the primary fungal genera, increased initially from day 7 to day 14 and then decreased from day 14 to day 35, reaching 39.14% at the end of fermentation. Its relative abundance was lowest on day 7 (7.80%) and highest on day 14 (47.60%). In contrast, *Thermoascus* decreased from day 7 to day 14, then increased from day 14 to day 40, peaking at 62.18% on day 35. Meanwhile, Rhizopus also showed an initial decrease (day 7–14) followed by an increase (day 14–21), with a maximum relative abundance of 8.64%. These filamentous fungi play significant roles in the saccharification of raw material starch [47,52]. Furthermore, studies by Tang et al. have established links between genera such as *Bacillus*, *Kroppenstedtia*, *Thermoascus*, and *Thermomyces* and the development of ammoniacal notes in *HTDQ* [49]. The following section will further analyze the microorganisms associated with the formation of key flavor compounds during *HTDQ* fermentation.

### 3.5. Correlation Between Key Flavor Compounds in HTDQ and Their Associated Microorganisms and Biosynthetic Pathways

Previously, we identified 14 key flavor compounds through which *HTDQ* influences the flavor of *Jiaomian* base baijiu. The formation of these compounds during *HTDQ* fermentation is closely associated with microbial activities. Therefore, to elucidate microbial contributions to these key flavor compounds, Spearman correlation analysis was performed between microbial taxa and the compounds. The results were then filtered based on significance (*p* < 0.05) and correlation strength (|r| > 0.7). Overall, bacterial communities during *HTDQ* fermentation showed predominantly positive correlations with the key flavor compounds, accounting for 77.78% of the correlation network. Specifically, five bacterial genera exhibited significant positive correlations with seven flavor compounds. Among the bacteria, *Thermoactinomyces* showed the strongest influence on key flavor compounds, displaying significant positive correlations with six compounds: 1-hexanol, 1-nonanol, furfural, furfuryl alcohol, hexanoic acid, and isoamyl alcohol. In addition, 1-nonanol was positively correlated with *Lactobacillus*. Ethyl hexanoate showed positive correlations with *Bromus_tectorum*, *Lactobacillus*, and *Saccharopolyspora*. Ethyl phenylacetate was also positively correlated with *Saccharopolyspora*. Furthermore, four bacterial genera showed significant negative correlations with two compounds. Furfuryl alcohol, hexanoic acid, and isoamyl alcohol were all positively correlated with *Candidatus_bacilloplasma* and *Thermoactinomyces*.

Further analysis of fungal correlations with key flavor compounds during *HTDQ* fermentation revealed that fungi were also predominantly positively associated with the compounds, representing 80.95% of the correlation network. Twelve fungal genera showed significant positive correlations with six flavor compounds. Among these, *Byssochlamys*, *Kazachstania, Leiothecium*, and *Trichothecium* exhibited the strongest associations with the key flavor compounds. For instance, *Byssochlamys* was positively correlated with four flavor compounds—ethyl decanoate, ethyl heptanoate, furfuryl alcohol, and hexanoic acid—while showing a negative correlation with isovaleric acid. Both *Kazachstania* and *Leiothecium* were positively correlated with five compounds: 1-nonanol, ethyl heptanoate, furfuryl alcohol, hexanoic acid, and isoamyl alcohol, and were negatively correlated with isovaleric acid. Similarly, *Trichothecium* showed positive correlations with 1-nonanol, furfuryl alcohol, hexanoic acid, and isoamyl alcohol, along with a negative correlation with isovaleric acid.

**Figure 5:**
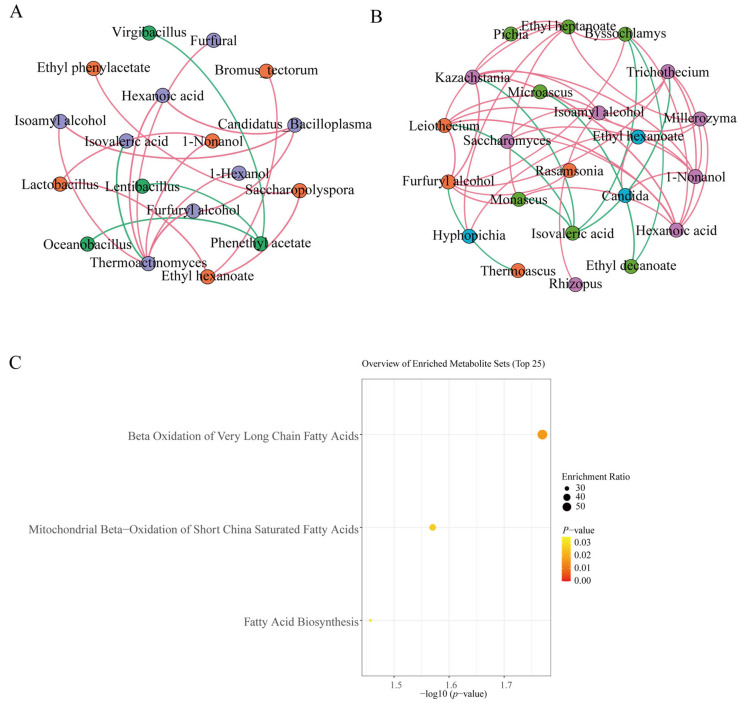
Analysis of microorganisms and metabolic pathways associated with the formation of key flavor compounds in *HTDQ*. (**A**) Bacteria, (**B**) fungi, and (**C**) associated pathways correlated with the formation of key flavor compounds. Red lines indicate positive correlations (*p* < 0.05, |r| > 0.7); Green lines indicate negative correlations (*p* < 0.05, |r| > 0.7). Thicker lines indicate stronger correlations.

To further explore the biosynthetic pathways of the key flavor compounds, metabolite enrichment analysis was performed. The results revealed that the formation of these metabolites is primarily associated with several core lipid metabolic pathways, including the beta oxidation of very long-chain fatty acids, mitochondrial beta-oxidation of short-chain saturated fatty acids, and fatty acid biosynthesis (Figure 5C). These pathways likely influence the formation of key flavor compounds by directly generating flavor molecules or their essential precursors. For instance, the beta oxidation of very long-chain fatty acids produces short-chain fatty acids, which can undergo esterification with alcohols to form various esters, a major class of aroma compounds. Furthermore, this oxidation process generates acetyl-CoA, a central precursor in the biosynthesis of numerous flavor substances [53]. Conversely, the fatty acid biosynthesis pathway utilizes acetyl-CoA to synthesize fatty acids. These fatty acids can, in turn, be oxidized when needed to generate flavor-active acids or other precursors, creating a dynamic metabolic cycle that supplies the building blocks for complex flavor profiles.

## 4. Conclusions

*Jiaomian* base baijiu is an important seasoning liquor in the blending of sauce-flavor baijiu, while *HTDQ* serves as a key source of characteristic aroma in base baijiu and significantly influences the final quality of sauce-flavor baijiu. To clarify the flavor compound basis of *HTDQ*’s influence on the flavor profiles of *Jiaomian* base baijiu, this study first conducted a comprehensive analysis of the aroma characteristics and flavor compound profiles between *Jiaomian* base baijiu and *Chuntian* base baijiu using sensory evaluation and flavor detection techniques. The results showed that *Jiaomian* base baijiu exhibited stronger “*Qu*” aroma and floral/fruity aroma. Six key flavor markers were identified to distinguish the two baijiu types: ethyl acetate, 1-propanol, acetaldehyde, furfural, propionic acid, and isoamyl alcohol. Subsequently, the aroma characteristics and flavor compounds of *HTDQ* itself were further analyzed. It was found that the differences in aroma characteristics between the surface and core layers were minor for white *HTDQ* and black *HTDQ*, while yellow *HTDQ* showed significant differences between its inner and outer layers, indicating strong heterogeneity. Further comparison of the aroma characteristics among the three *HTDQ* types revealed significant differences in both their inner and surface layers, primarily manifested as “*Qu*” aroma, sauce aroma, caramel aroma, sweet aroma, and musty notes. In-depth analysis of the flavor composition led to the identification of six differential markers, including tetramethylpyrazine, ethyl 3-phenylpropionate, and 2,3,5-trimethylpyrazine. Comparative analysis of the impact of different *HTDQ* types on *Jiaomian* base baijiu flavor revealed that yellow *HTDQ* exhibited the greatest heterogeneity between its inner and surface layers in terms of aroma profiles and flavor composition, contained the highest levels of flavor compounds, and exerted the strongest influence on the flavor of *Jiaomian* base baijiu.

By comparing flavor compounds between *HTDQ* and the two base baijiu types, 14 key compounds were identified that mediate *HTDQ*’s influence on *Jiaomian* base baijiu flavor. Tracing the dynamic changes in these key compounds during *HTDQ* fermentation showed that they were mainly formed in the early to middle fermentation stages. Correlation analysis further indicated that microorganisms during *HTDQ* fermentation were predominantly positively correlated with the key flavor compounds. Among them, *Thermoactinomyces, Byssochlamys, Kazachstania, Leiothecium*, and *Trichothecium* showed the closest associations—positively correlated with compounds such as 1-nonanol and furfuryl alcohol, and negatively correlated with isovaleric acid. Finally, KEGG enrichment analysis suggested that Beta Oxidation of Very Long-Chain Fatty Acids, Mitochondrial Beta Oxidation of Short-Chain Saturated Fatty Acids, and Fatty Acid Biosynthesis are key pathways involved in the formation of these flavor substances. In summary, this study clarifies the key flavor compounds through which different *HTDQ* types influence base baijiu flavor, reveals the microbial origins and metabolic pathways of these compounds, and provides a theoretical basis for regulating *HTDQ* production and improving base baijiu quality. These findings have important implications for the production and application of *HTDQ* and contribute to the high-quality production of *Jiaomian* base baijiu.

## Data Availability

The original contributions presented in the study are included in the article/Supplementary Materials. Further inquiries can be directed to the corresponding author.

## Supplementary Materials

The following supporting information can be downloaded at: https://www.mdpi.com/article/10.3390/foods15071124/s1, Figure S1: Temperature profile during the fermentation of High-Temperature Daqu; Table S1: RT of n-alkanes; Table S2: Calculation of RI; Table S3: References; Table S4: Identification result.

## Abbreviations

The following abbreviations are used in this manuscript:

HTDQ	High-Temperature Daqu
OPLS-DA	Orthogonal Partial Least Squares Discriminant Analysis
PCA	Principal Component Analysis
JMJ	Jiaomian base baijiu
CTJ	Chuntian base baijiu
HS-SPME-GC-MS	Headspace Solid-Phase Microextraction Gas Chromatography Mass Spectrometry
GC-FID	Gas Chromatography—Flame Ionization Detector
