# Supplementary figures and images for: Elucidating the Impact of High-Temperature Daqu on Base Baijiu of Sauce-Flavor Baijiu: From Key Aroma Compounds to Microbial Origins
Source: Foods. 2026 Mar 24;15(7):1124. doi: 10.3390/foods15071124 (PMC13073185; doi:10.3390/foods15071124)

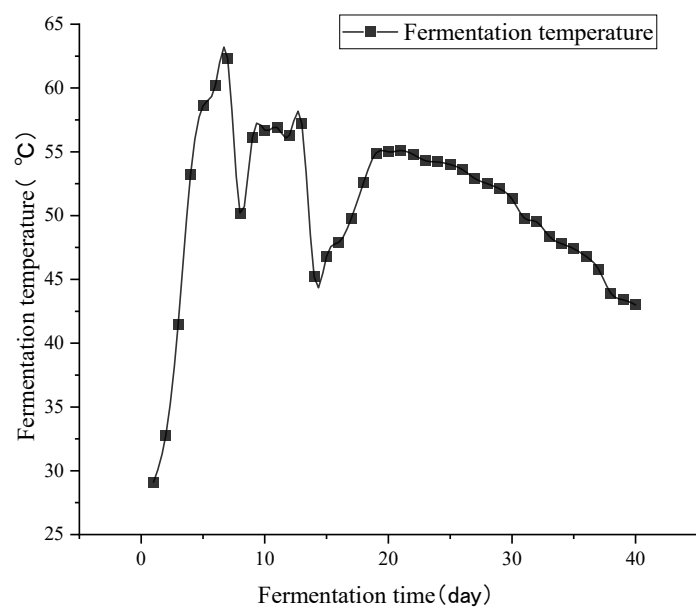

Figure S1. Temperature profile during the fermentation of High-Temperature *Daqu*

Supplement: Supplementary file 1 [file foods-15-01124-s001.zip › Figure S1.pdf]
